# Customized Prediction of Short Length of Stay Following Elective Cardiac Surgery in Elderly Patients Using a Genetic Algorithm

**DOI:** 10.4236/wjcs.2013.35034

**Published:** 2013-09

**Authors:** Joon Lee, Sapna Govindan, Leo A. Celi, Kamal R. Khabbaz, Balachundhar Subramaniam

**Affiliations:** 1 School of Public Health and Health Systems, University of Waterloo, Waterloo, Canada; 2 Harvard-MIT Division of Health Sciences and Technology, Cambridge, USA; 3 Beth Israel Deaconess Medical Center, Boston, USA

**Keywords:** Cardiac Surgery, Elderly, Length of Stay, Risk Prediction, Genetic Algorithm

## Abstract

**Objective:**

To develop a customized short LOS (<6 days) prediction model for geriatric patients receiving cardiac surgery, using local data and a computational feature selection algorithm.

**Design:**

Utilization of a machine learning algorithm in a prospectively collected STS database consisting of patients who received cardiac surgery between January 2002 and June 2011.

**Setting:**

Urban tertiary-care center.

**Participants:**

Geriatric patients aged 70 years or older at the time of cardiac surgery.

**Interventions:**

None.

**Measurements and Main Results:**

Predefined morbidity and mortality events were collected from the STS database. 23 clinically relevant predictors were investigated for short LOS prediction with a genetic algorithm (GenAlg) in 1426 patients. Due to the absence of an STS model for their particular surgery type, STS risk scores were unavailable for 771 patients. STS prediction achieved an AUC of 0.629 while the GenAlg achieved AUCs of 0.573 (in those with STS scores) and 0.691 (in those without STS scores). Among the patients with STS scores, the GenAlg features significantly associated with shorter LOS were absence of congestive heart failure (CHF) (OR = 0.59, p = 0.04), aortic valve procedure (OR = 1.54, p = 0.04), and shorter cross clamp time (OR = 0.99, p = 0.004). In those without STS prediction, short LOS was significantly correlated with younger age (OR = 0.93, p < 0.001), absence of CHF (OR = 0.53, p = 0.007), no preoperative use of beta blockers (OR = 0.66, p = 0.03), and shorter cross clamp time (OR = 0.99, p < 0.001).

**Conclusion:**

While the GenAlg-based models did not outperform STS prediction for patients with STS risk scores, our local-data-driven approach reliably predicted short LOS for cardiac surgery types that do not allow STS risk calculation. We advocate that each institution with sufficient observational data should build their own cardiac surgery risk models.

## 1. Introduction

With aging population, more and more geriatric patients with complex co-morbid conditions at increased risk for morbidity and mortality present for cardiac surgery [1]. Risk scores such as EuroSCORE [2] and the Society of Thoracic Surgeons (STS) risk score [3] are commonly used to prognosticate the risk of postoperative morbidity and mortality. However, these risk prediction models overestimate mortality risk, especially in high-risk and geriatric patients [4], which might lead to denial of surgery for deserving patients. Furthermore, these scores, having been derived from a large, heterogeneous population to optimize external validity, tend to perform well at the population level but not as well at individual level. Such sub-optimal predictive accuracy at the individual level could be attributed to the event (mortality) rates in the 10% - 15% range [5]. This event rate range is challenging to predict due to the computationally low prevalence but is clinically significant. In isolated aortic valve replacement (AVR) in octogenarians, the actual mortality rates were as low as 5% while the predicted rates by EuroSCORE and STS risk scores were three to four folds higher [6].

Morbidity is more common than mortality and usually leads to increased postoperative intensive care unit (ICU) length of stay (LOS) [7,8]. Increased postoperative ICU LOS leads to higher costs and hence is a concern in the presence of ever shrinking health care funds [9]. An accurate prediction of postoperative LOS allows better decision making, treatment triaging and better allocation of resources. Models such as EuroSCORE, originally constructed to assess mortality rates, have been subsequently used and validated for prediction of prolonged ICU stay [8]. Several models derived from different populations have attempted to predict prolonged ICU LOS. However, they had used different definitions for prolonged stay and did not specifically focus on high risk geriatric patients and/or short LOS stay [10-13]. Some scores such as STS cannot estimate risks for all patients undergoing cardiac surgery, as they have been derived and validated only for specific subsets of surgery such as coronary bypass grafting, valve surgery or a combination of both. As a result, the STS risk models are unable to provide a score for other types or combinations of cardiac surgery.

Therefore, there is a need for a patient-level risk prediction model for all patients undergoing cardiac surgery, especially for those at a high risk such as geriatric patients. We hypothesized that a local, custom built model derived from a more homogeneous subset of patients would more accurately predict morbidity risk in geriatric populations, thus enabling proactive decision making. In the present study, we employed a machine learning technique called the genetic algorithm (GenAlg) to develop a customized model for short post-surgery LOS prediction and compared its performance with that of the STS score.

## 2. Methods

### 2.1. Database

We performed a retrospective medical records review of geriatric patients who were aged 70 or older, and underwent elective cardiac surgery from January 2002 to June 2011 at an urban tertiary-care center. We obtained approval from the Institutional Review Board of our institution. We obtained the records from the Society of Thoracic Surgeons (STS) database maintained by trained personnel at our institution since 2001. Two trained cardiac surgical nurses report outcomes to the STS database in a quarterly fashion. They have periodic internal checks and also receive reports from the Department of Public Health liaison on a yearly basis commenting on data quality and necessary corrections. The database contains all preoperative (e.g., demographic data, co-morbid conditions, inotropic support, STS risk predictions for morbidity and mortality), intraoperative (e.g., cross clamp time), and postoperative adverse outcomes (e.g., STS predefined morbid events such as stroke, atrial fibrillation, renal failure, myocardial infarction, mortality, pulmonary morbidity, sternal infection, and prolonged LOS).

The STS predefined morbid events include [3]:

- Operative mortality: death during the same hospitalization as surgery regardless of timing, or within 30 days of surgery regardless of venue
- Permanent stroke (cerebrovascular accident): a central neurologic deficit persisting longer than 72 hours
- Renal failure: a new requirement for dialysis or an increase in serum creatinine to greater than 2.0 mg/dL and double the most recent preoperative creatinine level
- Prolonged ventilation: ventilation for more than 24 hours
- Deep sternal wound infection
- Reoperation for any reason
- Major morbidity or mortality: a composite defined as the occurrence of any of the above end points
- Prolonged postoperative LOS: LOS greater than 14 days (alive or dead)
- Short postoperative LOS: LOS less than 6 days and patient discharged alive

### 2.2. Feature Pool

The following 22 preoperative features (predictors) were investigated for their predictive power for short LOS (defined by STS as LOS < 6 days): age, gender, race, family history of coronary artery disease (CAD), diabetes mellitus (DM), hypertension (HTN), chronic lung disease, cerebrovascular disease (CVD), peripheral vascular disease (PVD), myocardial infarction (MI), congestive heart failure (CHF), preoperative use of the following medications: beta blockers, angiotensin converting enzyme inhibitors (ACEI), intravenous (IV) nitrates, anticoagulants, inotropes, steroids, and aspirin; the type of cardiac surgery: coronary artery bypass graft placement (CABG), aortic valve procedure, mitral valve procedure, and last creatinine measurement prior to the surgery. In addition, one intraoperative variable was added to the feature pool: cross clamp time. Hence, a total of 23 features were investigated.

### 2.3. Feature Selection and Evaluation

We applied a GenAlg [14] to computationally select features for prediction of short LOS. GenAlgs are optimization routines that attempt to find a set of features that maximizes a user-defined fitness function. In this study, the output of the fitness function was the area under the receiver operating characteristic curve (AUC) of a logistic regression (LR) model designed for short LOS prediction. Each combination of features was evaluated using the fitness function, and the entire population of feature sets advanced to the next generation with crossover and mutation in an attempt to “breed” fitter feature sets, which is analogous to the process of biological evolution. The population size (number of feature combinations) in this study was 1000. The entire data was randomly divided into 49% training, 21% validation, and 30% test data. While the training data was used to build an LR model for a given feature combination, the output of the fitness function was the predictive performance (measured in AUC) of the LR model on the validation data. The final AUCs for the selected features were computed based on the test data which had been held out prior to the execution of the GenAlg and were the same across different numbers of features.

The GenAlg was executed separately for varying numbers of selected features: 5, 10, 15, and 20. All features, 23 of them, were also evaluated without the GenAlg. Since GenAlg performance depends on the initial population of randomly selected feature sets, the GenAlg was repeated 5 times for each number of features. Among the 5 attempts, the feature combination corresponding to the highest AUC based on the validation data was selected for the final evaluation on the test data. Each GenAlg run was terminated after 50 generations.

STS prediction for short LOS was not available for all patients in our cohort. This is due to the fact that STS does not have a prediction model for every type of cardiac surgery. Therefore, the GenAlg was conducted independently on two sub-cohorts: the patients with an STS short LOS prediction and those without. STS prediction served as the gold standard in the sub-cohort where STS prediction was available, and STS AUC was computed on the same test data on which the GenAlgselected features were evaluated.

Lastly, we built an LR model on all patients (without training, validation, and test partitions) using the selected features in order to compute odds ratios (ORs) and the corresponding 95% confidence intervals (CI). However, this analysis was still conducted separately for the two sub-cohorts with and without STS prediction. Statistical significance was reached when p-value was less than 0.05.

All feature selection and evaluation were conducted in MATLAB version R2010b (Mathworks, Natick, MA, USA).

## 3. Results

1426 patients met the inclusion criteria and were analyzed. Table 1 summarizes the characteristics of the patient cohort, stratified by availability of STS short LOS prediction. There were slightly more patients without STS prediction than with. Short LOS accounted for 44.1% and 31.1% of those with and without STS prediction, respectively. The patients with STS prediction were slightly younger than those without, and the proportion of male gender was higher among the patients with STS prediction than among those without. Furthermore, the patients with STS prediction were generally in worse condition than those without, which is supported by the higher prevalence of co-morbidities and preoperative medication use. While an overwhelming majority of the patients with STS prediction underwent CABG (possibly along with other procedures), the valve procedures were more frequent among those without STS than those with.

Table 2 shows AUCs from test data associated with the features selected by the GenAlg. STS prediction achieved an AUC of 0.629 on the same test data. Overall, our GenAlg approach was unable to outperform STS for the patients with STS prediction. In general, higher AUCs were achieved for the cases without STS prediction than for those with STS prediction. Maximum AUCs of 0.573 and 0.691 were achieved with 15 and 10 features for the sub-cohorts with and without STS prediction, respectively.

Table 3 lists the selected features, along with their ORs and 95% CIs, for the patients with STS prediction. The features that were selected consistently across different numbers of features were preoperative use of aspirin, aortic valve procedure, creatinine, and cross clamp time. However, preoperative use of aspirin and creatinine never reached statistical significance in any of the LR models. In the 15 feature model that resulted in the highest AUC (see Table 2), short LOS was significantly correlated with absence of CHF (OR = 0.59, p = 0.04), aortic valve procedure (OR = 1.54, p = 0.04), and shorter cross clamp time (OR = 0.99, p = 0.004).

Table 4 tabulates the counterpart information of Table 3 for the patients without STS prediction. Age, preoperative use of IV nitrates, and perfusion time were consistently selected by the GenAlg throughout the various numbers of features. Preoperative use of IV nitrates was statistically insignificant in all models. As Table 2 showed, the 10 feature set achieved the maximum AUC and revealed that short LOS was significantly correlated with younger age (OR = 0.93, p < 0.001), absence of CHF (OR = 0.53, p = 0.007), no preoperative use of beta blockers (OR = 0.66, p = 0.03), and shorter cross clamp time (OR = 0.99, p < 0.001).

## 4. Discussion

Our objective was to assess whether a custom model would more accurately predict morbidity in comparison with the established STS model in our local geriatric population. We utilized a GenAlg, an evolutionary algorithm that can perform automated feature selection to maximize predictive performance. In addition to the preoperative variables already utilized in the STS score, we also used intraoperative variables as these variables also influence prognosis [15]. In the present study, the GenAlg-based model could not outperform the STS score in those subjects with STS data. While the STS scores achieved an AUC of 0.629 in those with STS prediction, our GenAlg-based model achieved a maximum AUC of 0.573 in the subset with STS prediction and a maximum AUC of 0.691 in those without. Also, it is worthwhile noting that the GenAlg-based model consistently achieved higher AUCs in those cases lacking an STS prediction than in those with STS prediction. Hence, GenAlg-based modeling was shown to be useful for predicting shorter LOS in geriatric patients for whom STS risk scores cannot be calculated. Furthermore, the GenAlg-based model demonstrated better prediction utilizing fewer variables (10 to 15), whereas the STS models use more than 30 variables. The significant association of shorter cross clamp time with short LOS indicates the influence of intraoperative parameters on postoperative course. It is important to note that intraoperative parameters are not part of the STS scoring system [16].

Accurate prediction of prolonged post-cardiac-surgery LOS can be a crucial piece of information for health care cost savings. The earlier clinicians and hospital administrators are informed of potential excessive consumption of hospital resources, the better they can allocate the limited resources they have. Moreover, keeping LOS at minimum is important with respect to managing risk for hospital-acquired infections.

Building customized models rather than the traditional one-model-fits-all approach has been shown to be meritorious in mortality prediction [17]. Such tailored clinical decision support is now possible largely thanks to the advent of electronic health data that led to the formation and maintenance of large local databases containing an enormous amount of patient information. With respect to decision support driven by local health data, the Institute of Medicine has recently elaborated on the need to start analyzing routinely collected local data during patient care in order to improve care processes as well as clinical outcomes [18].

In a systematic review and validation of prediction of prolonged LOS following cardiac surgery, Ettema *et al.* [8] found that the Parsonnet score [19] (AUC of 0.75) and EuroScore [20] (AUC of 0.71) were superior to the 20 models they chose to study. The focus in this particular study was prolonged ICU LOS. ICU stay can be a nebulous definition as different ICUs have different criteria for ICU care. In addition, their definition of prolonged ICU LOS was >48 hours of ICU stay. We chose to study hospital stay as an outcome and we focused on prediction of shorter LOS following cardiac surgery in high risk geriatric patients only.

STS is limited to three risk models—CABG, Valve, and CABG + Valve [3,16]. These risk models apply to seven types of surgery—CABG, aortic valve replacement (AVR), mitral valve replacement (MVR), mitral valve repair (MV Repair), CABG + AVR, CABG + MVR, and CABG + MV Repair. An STS risk score cannot be calculated for any procedure that does not precisely fall into any of these categories. Also, age and gender are required variables; no risk score can be calculated if either is not known. Our GenAlg approach performed better at short LOS prediction among the patients without STS data compared to those with. The model performance is based on the ability to discriminate between those with and without short LOS and is expressed as an AUC. An AUC of 1 correlates with perfect prediction and that of 0.5 translates to no predictive ability or leaving it to chance. An AUC < 0.7 should be applied in clinical practice with caution. The GenAlg-based model achieved a maximum AUC of 0.691 in those without STS prediction. The better discriminating ability of our local model in those lacking STS risk scores points to the utility of this model for such patients. Further studies are required to confirm this effect in similar patient groups. The discriminative ability of a model not only depends on the model itself but also on the dataset or population it is tested on [21]. One of the known weaknesses of AUC is that it overestimates performance in a skewed data set. Furthermore, the larger sample size of the sub-cohort without STS prediction could have been a factor in the improved performance.

One area for future work is to validate our customized GenAlg-driven risk modeling approach (rather than our specific models since they were customized for our institution) at other institutions for external validity. Ultimately, an impact study will have to be conducted to gauge the benefits of having accurate LOS prediction for cardiac patients with respect to cost savings and reduction of hospital-acquired infections.

## 5. Conclusion

Our GenAlg-based models did not outperform STS prediction for patients with STS risk scores. However, our customized approach based on local data reliably predicted short LOS for cardiac surgery types that do not allow STS risk calculation. The primary strength of our proposed risk stratification is its utilization of the most relevant data from a local data repository rather than one-size-fits-all models. We advocate that each institution with sufficient observational data should build their own risk models.

## Figures and Tables

**Table 1:** Patient cohort characteristics, stratified by availability of STS short LOS prediction.

	STS prediction available	STS prediction unavailable	p-value^2^
Number of patients	655	771	
Post-surgery LOS (days)^1^	6 [5, 8]	7 [5, 9]	<0.001
Post-surgery LOS < 6 days	44.1%	31.1%	<0.001
Age (years)^1^	76 [72, 80]	77 [73, 81]	<0.001
Gender (male)	67.6%	54.0%	<0.001
Race (non-Caucasian)	5.2%	4.3%	0.42
Family history of CAD	16.8%	14.9%	0.33
Diabetes mellitus	35.0%	25.0%	<0.001
Hypertension	87.2%	83.5%	0.05
Chronic lung disease	9.5%	16.1%	<0.001
CVD	17.4%	21.9%	0.03
PVD	17.7%	16.5%	0.54
MI	29.0%	19.7%	<0.001
CHF	15.6%	23.7%	<0.001
Preoperative use of beta blockers	76.8%	70.7%	0.009
Preoperative use of ACEI	47.3%	40.2%	0.007
Preoperative use of IV nitrates	3.4%	1.7%	0.04
Preoperative use of anticoagulants	20.0%	24.1%	0.06
Preoperative use of inotropes	0%	0.1%	0.36
Preoperative use of steroids	3.4%	4.3%	0.37
Preoperative use of aspirin	87.2%	70.9%	<0.001
CABG	80.6%	43.3%	<0.001
Aortic valve procedure	23.4%	54.3%	<0.001
Mitral valve procedure	7.0%	25.3%	<0.001
Last pre-surgery creatinine (mg/dL)^1^	1.0 [0.9, 1.2]	1 [0.8, 1.3]	0.88
Cross clamp time (min)^1^	63 [49, 75]	80 [63, 104]	<0.001

LOS: length of stay, CAD: coronary artery disease, CVD: cerebrovascular disease, PVD: peripheral vascular disease, MI: myocardial infarction, CHF: congestive heart failure, ACEI: angiotensin converting enzyme inhibitors, IV: intravenous, CABG: coronary artery bypass graft;

1 median [Q1, Q3],

2 calculated via the chi-squared test for binary variables and the Wilcoxon rank sum test for continuous variables.

**Table 2:** Short LOS prediction AUC using the genetic algorithm and logistic regression. Different numbers of selected features are shown for the two sub-cohorts: those with and without STS prediction. For comparison, the STS prediction model achieved an AUC of 0.629 for the patients with calculated STS scores for short LOS.

Number of features	STS prediction available	STS prediction unavailable
5	0.545	0.681
10	0.563	0.691
15	0.573	0.676
20	0.551	0.672
23 (all features)	0.555	0.680

**Table 3:** Features selected by the genetic algorithm for short LOS prediction among the patients who had STS predictions available. For each number of features, only the selected ones are shown with OR and 95% CI. The corresponding AUCs on test data are tabulated in Table 2.

Features	Number of features			

	5	10	15	20
Age (yr)				
Gender (male)			1.00 (0.69 to 1.44)	1.07 (0.74 to 1.56)
Ethnicity (non-Caucasian)			1.03 (0.49 to 2.18)	1.02 (0.48 to 2.16)
Family history of CAD			0.95 (0.61 to 1.48)	
Diabetes mellitus				1.02 (0.71 to 1.46)
Hypertension			0.67 (0.41 to 1.09)	0.71 (0.43 to 1.16)
Chronic lung disease		1.08 (0.61 to 1.93)		
CVD			0.88 (0.56 to 1.39)	0.90 (0.57 to 1.43)
PVD		0.73 (0.47 to 1.15)	0.75 (0.47 to 1.19)	0.79 (0.49 to 1.26)
MI		0.79 (0.54 to 1.15)		0.88 (0.60 to 1.30)
CHF			0.59* (0.36 to 0.97)	0.57* (0.34 to 0.94)
Preoperative beta blockers	0.76 (0.51 to 1.13)	0.80 (0.53 to 1.21)		0.85 (0.56 to 1.30)
Preoperative ACEI			0.99 (0.71 to 1.38)	0.96 (0.68 to 1.35)
Preoperative IV nitrates			0.62 (0.23 to 1.67)	0.72 (0.27 to 1.94)
Preoperative anticoagulants			0.95 (0.61 to 1.47)	0.88 (0.56 to 1.40)
Preoperative inotropes		1.00 (1.00 to 1.00)		1.00 (1.00 to 1.00)
Preoperative steroids			1.75 (0.68 to 4.48)	1.80 (0.70 to 4.64)
Preoperative aspirin	1.40 (0.85 to 2.30)	1.46 (0.88 to 2.42)	1.33 (0.80 to 2.21)	1.48 (0.88 to 2.51)
CABG		0.85 (0.50 to 1.45)		1.08 (0.55 to 2.15)
Aortic valve procedure	1.50* (1.00 to 2.24)	1.29 (0.78 to 2.13)	1.54* (1.02 to 2.33)	1.73 (0.91 to 3.30)
Mitral valve procedure				2.16 (0.90 to 5.18)
Creatinine (mg/dl)	0.80 (0.62 to 1.04)	0.82 (0.64 to 1.06)	0.83 (0.65 to 1.07)	0.84 (0.65 to 1.08)
Perfusion time (min)	0.99* (0.98 to 1.00)	0.99* (0.98 to 1.00)	0.99* (0.98 to 1.00)	0.99* (0.98 to 1.00)
				

OR: odds ratio, CI: confidence interval, CAD: coronary artery disease, CVD: cerebrovascular disease, PVD: peripheral vascular disease, MI: myocardial infarction, CHF: congestive heart failure, ACEI: angiotensin converting enzyme inhibitors, IV: intravenous, CABG: coronary artery bypass graft;

* Statistically significant (p < 0.05).

**Table 4:** Features selected by the genetic algorithm for short LOS prediction among the patients who did not have STS predictions available. For each number of features, only the selected ones are shown with OR and 95% CI. The corresponding AUCs on test data are tabulated in Table 2.

Features	Number of features			

	5	10	15	20
Age (yr)	0.93* (0.89 to 0.96)	0.93* (0.90 to 0.97)	0.93* (0.89 to 0.96)	0.94* (0.90 to 0.98)
Gender (male)				1.56* (1.05 to 2.32)
Ethnicity (non-Caucasian)			0.48 (0.15 to 1.51)	0.44 (0.13 to 1.44)
Family history of CAD		1.30 (0.81 to 2.10)	1.26 (0.77 to 2.04)	1.19 (0.73 to 1.95)
Diabetes mellitus				1.15 (0.74 to 1.78)
Hypertension				0.70 (0.43 to 1.13)
Chronic lung disease		1.07 (0.64 to 1.77)	1.04 (0.63 to 1.72)	1.16 (0.70 to 1.94)
CVD				0.69 (0.43 to 1.12)
PVD				0.75 (0.42 to 1.33)
MI			0.97 (0.60 to 1.57)	
CHF		0.53* (0.33 to 0.84)	0.58* (0.36 to 0.94)	0.59* (0.37 to 0.97)
Preoperative beta blockers		0.66* (0.45 to 0.96)	0.65* (0.44 to 0.97)	
Preoperative ACEI		1.36 (0.95 to 1.96)	1.41 (0.97 to 2.03)	1.43 (0.98 to 2.10)
Preoperative IV nitrates	0.27 (0.03 to 2.29)	0.36 (0.04 to 3.09)	0.33 (0.04 to 2.90)	0.32 (0.03 to 2.91)
Preoperative anticoagulants			1.07 (0.68 to 1.68)	1.00 (0.64 to 1.57)
Preoperative inotropes		1.00 (1.00 to 1.00)	1.00 (1.00 to 1.00)	1.00 (1.00 to 1.00)
Preoperative steroids		0.81 (0.26 to 2.56)		
Preoperative aspirin	1.16 (0.78 to 1.71)		1.19 (0.79 to 1.80)	1.14 (0.75 to 1.73)
CABG				1.07 (0.70 to 1.63)
Aortic valve procedure				0.85 (0.53 to 1.37)
Mitral valve procedure	0.63* (0.41 to 0.95)		0.69 (0.45 to 1.06)	0.59* (0.34 to 1.00)
Creatinine (mg/dl)			0.91 (0.59 to 1.40)	0.82 (0.50 to 1.35)
Perfusion time (min)	0.99* (0.98 to 0.99)	0.99* (0.98 to 0.99)	0.99* (0.98 to 0.99)	0.99* (0.98 to 0.99)

OR: odds ratio, CI: confidence interval, CAD: coronary artery disease, CVD: cerebrovascular disease, PVD: peripheral vascular disease, MI: myocardial infarction, CHF: congestive heart failure, ACEI: angiotensin converting enzyme inhibitors, IV: intravenous, CABG: coronary artery bypass graft;

* Statistically significant (p < 0.05).
